# Are Solid Particles Ready for Prime-Time Proteomics?

**DOI:** 10.1021/acs.analchem.5c03073

**Published:** 2025-08-20

**Authors:** Eduardo S. Kitano, Yana Demyanenko, Shabaz Mohammed

**Affiliations:** † 590430 Rosalind Franklin Institute, Harwell Campus, OX11 0QX Didcot, United Kingdom; ‡ Department of Pharmacology, 6396 University of Oxford, OX1 3QT Oxford, United Kingdom; § Department of Biochemistry, University of Oxford, OX1 3QU Oxford, United Kingdom; ∥ Department of Chemistry, University of Oxford, OX1 3TA Oxford, United Kingdom

## Abstract

We evaluate the performance of nonporous C-18 stationary phases in high-speed proteomics workflows. We employed two commercially available sub-2 μm nonporous particle (NPP) materials, ODS-IIIE (1.5 μm) and SOLAD (1.0 μm), to fabricate analytical columns using 150 μm internal diameter (i.d.) fused silica capillaries to ensure compatibility with nano-UHPLC and nano-HPLC pressure regimes. Using “long” NPP columns (15–25 cm) connected to a conventional nano-UHPLC, we found that both materials supported efficient peptide separations within the flow rate and pressure ranges typical of nano-UHPLC systems. Shorter columns, used with the 10- and 16 min Whisper Zoom 120 and Zoom 80 methods on the Evosep One HPLC, demonstrated competitive separation performance compared to columns packed with fully porous (FPP) and superficially porous particles (SPP), achieving full-width at half-maximum (FWHM) values below 2 s (Zoom 120) and 3 s (Zoom 80). DIA LC-MS/MS analysis of a human cell lysate digest demonstrated that NPP columns provided performance comparable to or slightly superior to those of FPP and SPP materials. These findings establish NPP-based columns as a viable and competitive alternative to FPP and SPP materials, particularly suited for high-throughput and high-sensitivity proteomics applications.

## Graphical abstract

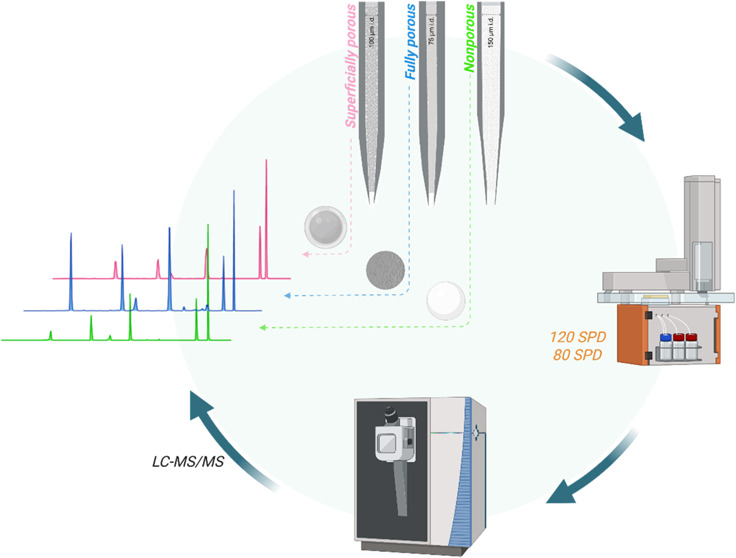

## Introduction

Recent technological advancements in mass spectrometry (MS) have significantly increased sensitivity and acquisition speeds,^1,2^ reshaping the design and optimization of liquid chromatography–mass spectrometry (LC-MS) workflows.^3–6^ As a result, short-gradient LC-MS/MS methods, particularly when integrated with data-independent acquisition (DIA) strategies, are increasingly employed in high-throughput applications such as clinical proteomics,^7,8^ biomarker discovery,^9,10^ and single-cell proteomics.^11–13^

Underlying much of this progress is the widespread adoption of sub-2 μm fully porous C-18 particles (FPPs) in column design, which have proven crucial in proteomics due to their high surface area, excellent mass transfer properties, and broad compatibility with both nano- and microflow systems.^14,15^ While FPPs remain the dominant stationary phase, alternative technologies such as superficially porous particles (SPPs) and nonporous particles (NPPs) have shown considerable potential for LC-MS/MS applications.^16–18^ Both SPP and NPP C-18 phases offer potentially distinct advantages over FPPs, including superior mass transfer kinetics, reduced peak dispersion, and higher resolution at high flow rates—features that are particularly advantageous for high-speed separations.^19–22^ Due to their inherently low surface area, nonporous particles have limited sample-loading capacity, restricting their application to low sample amounts^23^ while the higher surface area of the porous shell in SPPs enables loading capacities comparable to those of FPPs.^24^ The enhanced chromatographic performance of these materials results in sharper peaks^23^ and stronger signal intensity, which in data-dependent acquisition (DDA) improves precursor ion selection, and in DIA enhances the separation of coeluting species, leading to cleaner MS/MS spectra and more confident peptide identification and quantification.^25,26^ The potential higher-separation power of these materials positions both SPP and NPP as attractive tools for the next generation of fast and highly sensitive proteomic workflows.

Although the use of NPP-based columns in proteomics has been limited,^25^ the subnanogram sample requirements of modern mass spectrometers now position them as a compelling option for high-efficiency separations in low-input proteomic applications. In this study, we investigate the applicability of NPP and SPP columns to high-throughput proteomic workflows. Separation performance was evaluated using the two newly established short-gradient methods on an Evosep One LC system and compared to established C-18 materials employed in peptide separation. Coupled with DIA, we compared the peptide and protein identification efficiency of short NPP and SPP columns to those of FPP columns using a human cell lysate.

## Experimental Section

Detailed methods are provided in the Supporting Information.

### Protein Digestion and Sample Preparation

Bovine serum albumin (BSA) and α-casein proteins (Sigma), and Expi 293F cell (Gibco) lysate were submitted to in-solution LysC/trypsin digestion as previously described.^27^ The resulting peptide samples were resuspended in either 5% formic acid/5% DMSO for nano-UHPLC analysis or in 5% formic acid/0.015% *n*-Dodecyl-β-D-maltoside (DDM) prior to loading onto Evotip pure (Evosep Biosystems) for subsequent nano-HPLC analysis.

### Column Packing

Nonporous, fully porous, and superficially porous C-18 materials were prepared as slurries at a concentration of 100 mg/mL. Specifically, Reprosil Gold (fully porous), ODS-IIIE (nonporous), and SOLAD (nonporous) were slurried in 100% acetone, while Luna Omega Polar (fully porous) and Kinetex (superficially porous) were slurried in 100% methanol. Fused silica capillaries (Polymicro Technologies) with internal diameters (i.d.) of 75, 100, and 150 μm were pulled by using a P-2000 laser puller (Sutter Instrument). These capillaries were then packed with the respective C-18 materials using a PC8500 Pressure Injection Cell (Next Advance), following the protocol described by Kovalchuck,^28^ with the production of self-assembled particles as a frit at the outlet of the columns.^29^ Seventy-five micrometer i.d. capillaries were packed with fully porous Reprosil Gold (1.9 μm, Dr. Maisch) and Luna Omega Polar (1.6 μm, Phenomenex); a 100 μm i.d. capillary with superficially porous Kinetex (1.7 μm, Phenomenex); and 150 μm i.d. capillaries with nonporous ODS-IIIE (1.5 μm, Eprogen/Promigen Life Sciences) and SOLAD (1.0 μm, Glantreo). Following packing, the column beds were consolidated by applying a constant flow of 70% acetonitrile at 800 bar for 2 h. Columns were then cut to the desired length, and a sol–gel frit was created at the outlet according to the method described by Maiolica.^30^ Figure 1 schematically illustrates the different C-18 materials and the columns fabricated in this study.

**Figure 1:**
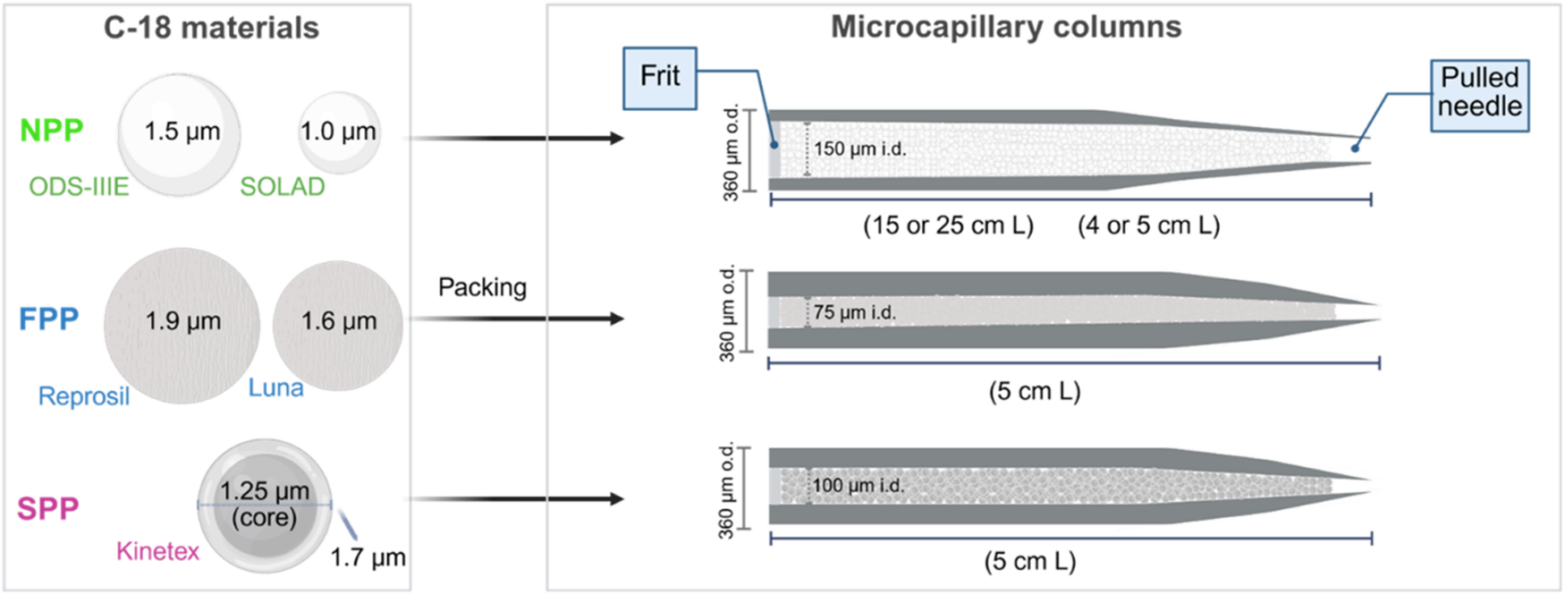
Schematic representation of the C-18 stationary phases and column configurations employed in this study. The left panel presents the morphological characteristics of nonporous (NPP), fully porous (FPP), and superficially porous (SPP) C-18 particles, with particle sizes represented proportionally. The right panel summarizes the key specifications of the columns packed with each respective particle type.

### Nano-Liquid Chromatography and Mass Spectrometry

To optimize flow rates and column temperatures for the NPP C-18 columns, 10 fmol of BSA/casein digest was separated using an Ultimate 3000 RSLCnano system (Thermo Fisher Scientific), separated on 25 cm ODS-IIIE and 15 cm SOLAD analytical columns. The chromatographic separations were performed across a temperature range of 30 to 70 °C (at a constant flow rate of 200 nL/min), and at flow rates ranging from 50 to 500 nL/min at 40 °C (ODS-IIIE) and 50 °C (SOLAD), utilizing a 15 min linear gradient of 7–55% solvent B (5% DMSO, 0.1% formic acid in acetonitrile). The BSA/casein digest was also subjected to a 15 min separation using shorter ODS-IIIE (5 cm) and SOLAD (4 cm) columns at 200 nL/min at 20 °C. To assess and compare the separation performance of NPP columns with that of FPP and SPP columns, a 10 fmol BSA/casein digest was analyzed using the predefined Whisper Zoom 120 (10 min gradient) and Zoom 80 (16 min gradient) methods on an Evosep One HPLC system (Evosep Biosystems). Increasing amounts of Expi 293F digest (0.25 to 50 ng) separated by the Whisper Zoom 120 and Zoom 80 methods were used for the assessment of peptide loading capacity of 5 cm ODS-IIIE and 4 cm SOLAD columns. Data-dependent acquisition (DDA) mass spectrometry analyses were performed on a Q Exactive mass spectrometer (Thermo Fisher Scientific).

Twenty nanograms of Expi 293F digest was separated on NPP, FPP, and SPP C-18 columns using the Whisper Zoom 120 and Zoom 80 methods of the Evosep One coupled to an Orbitrap Exploris 480 mass spectrometer (Thermo Fisher Scientific) operating in data-independent acquisition (DIA) mode.

### Raw Data Analysis

Full-width at half-maximum (FWHM) and peptide intensities were manually calculated and obtained from the extracted ion chromatograms (XICs) of selected peptides from BSA/casein or Expi 293F digests using Freestyle software (Thermo Fisher Scientific). Peak capacity (Pc) was calculated using the method described by Kovalchuck.^28^ FWHM values were further processed using Graphpad Prism software and analyzed using one-way analysis of variance (ANOVA) followed by Tukey’s post hoc test to evaluate pairwise differences between groups. DIA LC-MS/MS data were analyzed by DIA-NN software^31^ using library-free search. Spectral library was predicted in silico from the Uniprot human proteome database (Proteome ID: UP000005640, downloaded in August 2022, 79,759 sequences). Methionine oxidation and cysteine carbamidomethylation were set as variable and fixed modification, respectively. Enzyme specificity was set to trypsin/P, up to one missed cleavage, and a minimum peptide length of 7 amino acids was allowed per peptide. Both the precursor and protein FDRs were filtered to 1%. ANOVA was used to determine whether there were statistically significant differences among the means of the peptide/protein IDs. The LC and MS method details are described in Supporting Information. Raw files and results from this study have been deposited to the ProteomeXchange Consortium via the PRIDE^32^ partner repository with the data set identifier PXD064019.

## Results and Discussion

### Evaluation and Optimization of Nonporous C-18 Columns for NanoLC Peptide Separation

MS sequencing speed improvements have created the need to generate high peak capacity and short LC gradients with equally short loading and regeneration times. Such criteria require shorter columns than those typically utilized in proteomics, where longer columns and longer gradients are *de rigueur*.^33,34^ For such experiments, 5 cm columns are the common length as found on Evosep One systems. We wanted to explore the role of particle type and its size at such column lengths. The use of smaller particles in chromatographic separations results in higher column efficiency,^20^ which can further reduce column length to minimize dead volume and potentially improve peak capacity. However, smaller particles also lead to an increase in backpressure, which might require the use of ultrahigh-pressure LC (UHPLC)^35^ or shorter than desired columns to manage the increased diffusional resistance. Given the favorable mass transfer kinetics^21^ and high chromatographic efficiency of sub-2 μm nonporous C-18 particles across a broad range of linear velocities,^36^ we hypothesize that these materials are promising candidates for the development of rapid and highly sensitive proteomics methods.

In this study, we chose two commercially available nonporous C-18 materials: ODS-IIIE and SOLAD, which have particle diameters of 1.5 and 1.0 μm, respectively (Figure 1). The smaller particle size of SOLAD is expected to enhance efficiency, but also to increase column backpressure. Preliminary experiments indicated that the relationship between optimal linear velocity and flow rate differs between nonporous and porous particles. Typical flow rates of nanoLC systems are in the 100–400 nL/min range. To ensure compatibility with nanoLC while evaluating performance characteristics specific to nonporous particle (NPP) columns, we selected a 150 μm internal diameter (i.d.) capillary as the starting format. Tip dimensions obtained from the 75 μm and 100 μm i.d. capillaries were slightly narrower compared to those from the 150 μm i.d. capillaries (Figure S1). Nevertheless, with mean values of 8.1 μm (i.d.) and 9.6 μm (o.d.) (standard deviations below 10% for both measurements) for 150 μm, these tip dimensions are appropriate for the expected flow rate. Using this configuration, we prepared 15 and 25 cm columns packed with SOLAD and ODS-IIIE stationary phases, respectively. A 15 min gradient was applied using a BSA and casein digest standard to assess optimal flow rate, temperature, and the associated pressure profiles. The separation window and FWHM values of selected peptides were used as a metric for chromatography performance. As expected, system pressure increases linearly with the increase in flow rate at a constant temperature (Figure 2A, and Table S1). Even with the reduction in the column length and higher temperature, SOLAD generated higher backpressures than a significantly longer ODS-IIIE column (15 cm versus 25 cm) at flow rates above 150 nL/min. To mitigate the increased pressure associated with higher flow rates, the column temperature was set to 75 °C for flow rates exceeding 350 nL/min (SOLAD) and 400 nL/min (ODS-IIIE). Under these conditions, we observed a compression of the elution window and a loss of resolution, consistent with the reduced residence time of the peptides due to the higher mobile phase volume passing through the column per unit time (Figures S2 and S3). Optimal separations were observed between 150 and 350 nL/min. We chose to focus on 200 nL/min, which is the typical flow rate used for nanoLC-MS. Across the tested temperature range, the ODS-IIIE column exhibited lower FWHM values than the SOLAD column (Figure 2B and Table S1). For the ODS-IIIE column, FWHM remained relatively consistent, ranging from 2.3 to 2.5 s, corresponding to peak capacities (Pc) of 231 and 213, respectively, with the lowest value observed at 40 °C. In contrast, the SOLAD column exhibited greater variability in FWHM (3.7 to 4.6 s, corresponding to Pc of 143 and 117, respectively) across the same temperature range, suggesting a more pronounced temperature effect on peak width compared to the ODS-IIIE column. The SOLAD column’s lowest FWHM was observed at 50 °C (3.7 s, Pc = 143). Under optimal conditions, ODS-IIIE and SOLAD showed similar chromatographic profiles and FWHM of 2.3 s (Pc = 231) and 3.7 s (Pc = 166), respectively (Figure 2C–D). These findings support the use of a 150 μm i.d. column as a suitable option for implementation in nanoLC systems, offering compatibility with the pressure constraints at typical flow rate regimes.

**Figure 2:**
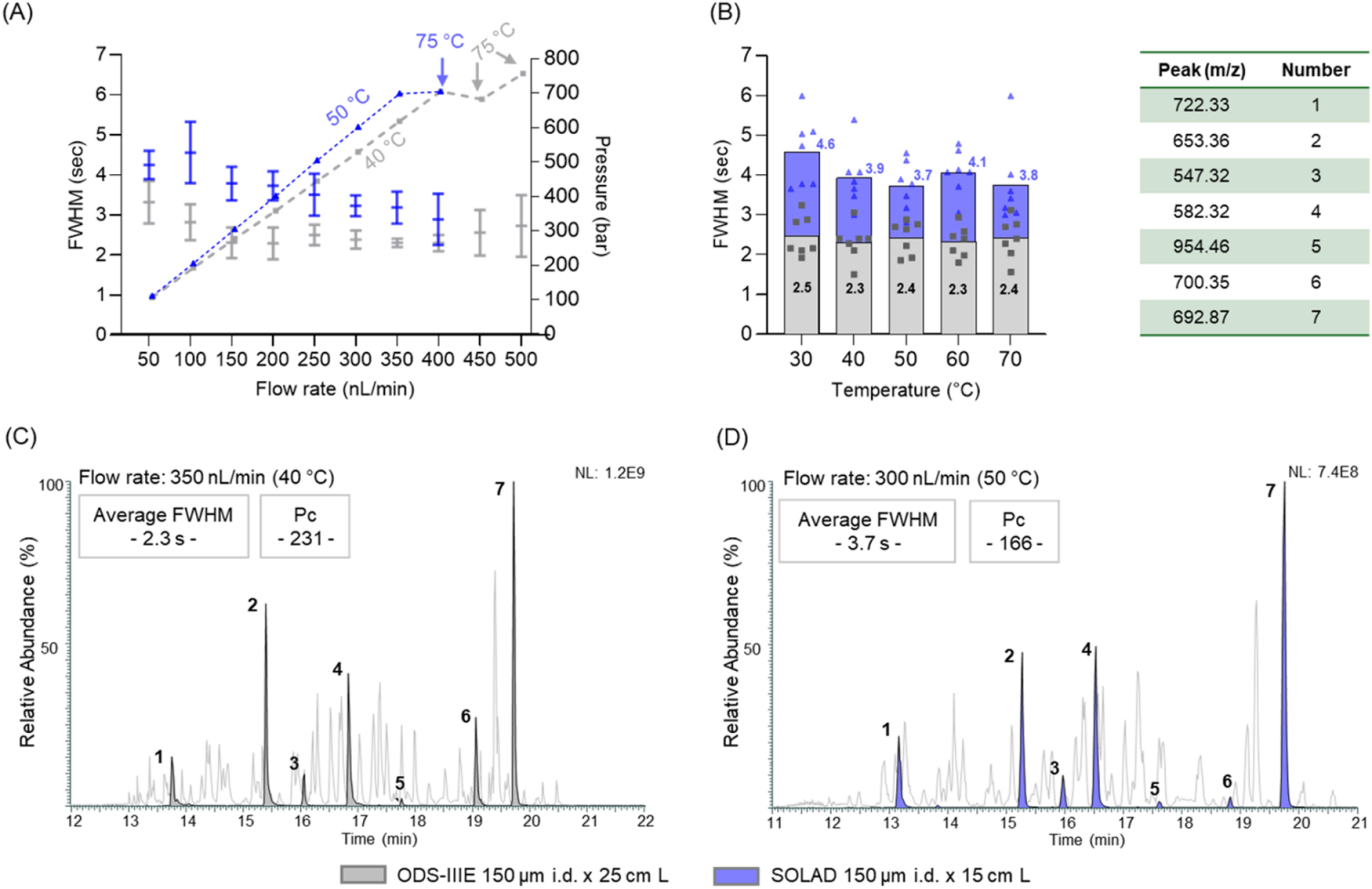
Optimization of nonporous particle columns for peptide separation. Average full-width at half-maximum (FWHM) of seven peptides from a 10 fmol bovine serum albumin/α-casein tryptic digest, separated using a 15 min gradient, was assessed across varying flow rates (A) using 150 μm i.d. × 25 cm ODS-IIIE and 15 cm SOLAD columns, and across varying column temperatures (B). Error bars indicate standard deviation (*n* = 3). Representative base peak (BPCs) and extracted ion chromatograms (XICs) under optimized conditions are displayed for the ODS-IIIE (C) and SOLAD (D) columns, along with their respective peak capacity (Pc) values. Peptide peaks are represented by the mass-to-charge ratio (*m*/*z*) shown in the table. LC-MS/MS analysis was performed on an Ultimate 3000 RSLCnano system coupled to a Q Exactive mass spectrometer.

### Short Nonporous C-18 for Fast Liquid Chromatography

We hypothesized that the Evosep One, with its newly developed Whisper Zoom methods operating at 200 nL/min, could be a suitable platform for the evaluation of NPP columns in fast LC-MS/MS. To ensure compatibility with the predefined methods and the pressure limits of the HPLC system, we chose a 5 cm column packed with ODS-IIIE particles, and given the higher backpressure associated with the smaller SOLAD particles, a shorter 4 cm column was selected. First, using an Ultimate LC, we conducted 15 min LC-MS/MS analyses of a BSA and casein digest at 200 nL/min using both long and short ODS-IIIE and SOLAD columns (Figure S4). Compared to longer columns, the shorter ODS-IIIE and SOLAD columns exhibited, as expected, significantly broader peaks and increased tailing across the entire elution range. This included early eluting, known BSA hydrophilic peptides with mass-to-charge ratio (*m*/*z*) 488.54, 625.78, and 722.33, which can be used as markers to assess the separation of hydrophilic species. This phenomenon is likely attributable to the mismatch in analyte retentivity between the trap and the analytical columns, which induces band broadening^25,37^ that is exacerbated by reduced column efficiency caused by shortening the analytical column. In the Evosep system, elution from the trap (Evotip) is diluted prior to reaching the analytical column, enabling reconcentration^3^ and thereby minimizing the impact of any selectivity mismatch. Next, we assessed the performance of the two short NPP columns on the Evosep system using the Whisper Zoom 120 (10 min) and Zoom 80 (16 min) methods, employing the same standard sample (Figure 3). A significant reduction in peak tailing and broadening was observed for both NPP columns under both methods. This suggests that the selectivity mismatch between the trap and analytical columns was indeed a primary cause of the previously observed deterioration in separation performance. However, hydrophilic peptides still exhibited poor resolution on both columns. Short ODS-IIIE and SOLAD column backpressures were below 100 bar at 200 nL/min on the Evosep LC (Figure S5), which is typical for the Whisper methods. Despite the utilization of columns with a diameter twice that of the standard 75 μm dimension, peptide elution was observed within the expected chromatographic range for both preset gradient methods, suggesting that the combination of higher density packing (lower dead volume) and enhanced mass transfer kinetics of nonporous particles effectively compensated for the increased column volume.

**Figure 3:**
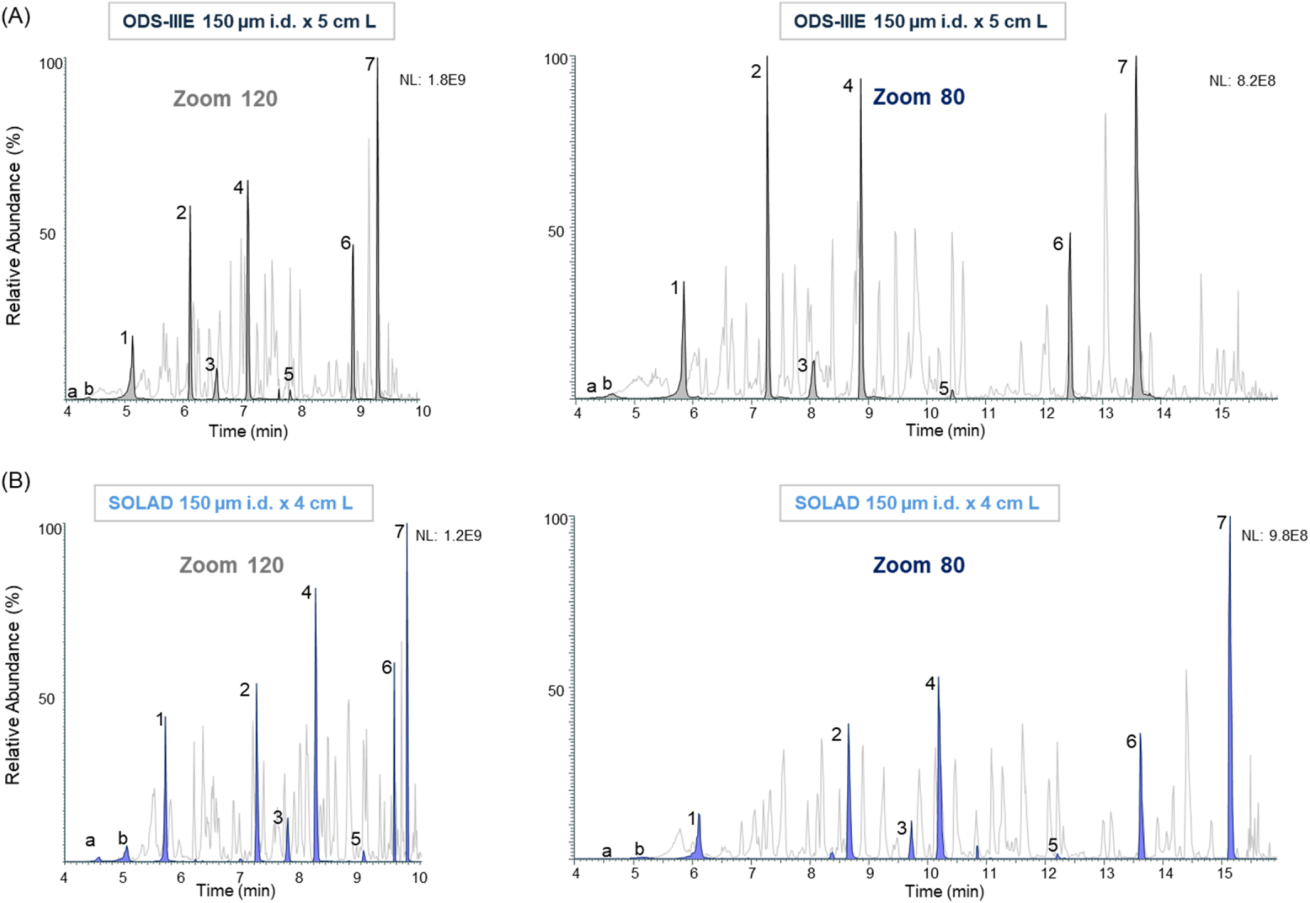
BPCs and XICs of selected tryptic peptides from a 10 fmol bovine serum albumin/α-casein digest separated on a 150 μm i.d. × 5 cm ODS-IIIE column (A) and a 150 μm i.d. × 4 cm SOLAD column (B) using Whisper Zoom 120 (left) and Zoom 80 (right) methods on an Evosep One LC system. Mass spectrometry detection was performed using a Q Exactive instrument.

### Performance Evaluation of Short Nonporous and Porous C-18 Columns for Peptide Separation in Fast Liquid Chromatography

Having established optimal operating conditions for the NPP columns and their feasibility for use with an Evosep LC, we proceeded to compare the separation performance of the short nonporous ODS-IIIE and SOLAD columns with three commonly utilized C-18 materials for peptide separation: a 1.6 μm FPP Luna Omega Polar (75 μm i.d. × 5 cm), a 1.9 μm Reprosil Gold (75 μm i.d. × 5 cm), and a 1.7 μm [1.25 μm (core) and 0.23 μm (shell thickness)] SPP Kinetex (100 μm i.d. × 5 cm), all designed to work at similar pressure range on the Evosep system (Figures 1 and S4). Tip dimensions obtained from the 75 μm and 100 μm i.d. capillaries were slightly smaller compared to those from the 150 μm i.d. capillaries (Figure S1). We conducted LC-MS/MS analyses of the same standard digest using the ODS-IIIE, SOLAD, Luna, Reprosil, and Kinetex columns with the Whisper Zoom 120 and Zoom 80 methods, and the separation performance of each column was evaluated by assessing the FWHM values of the seven selected peptide peaks (Figure 4 and Table S2). Visual inspection of the base-peak and extracted ion chromatograms (Figure 4A) revealed similar elution profiles across all columns under both gradient methods. As expected for the longer gradient, Zoom 80 exhibited broader and more widely spaced peaks. Differences in elution times for the same peptide across different columns, observed in both methods, likely arise from variations in stationary phase chemistries and/or column volumes. At Zoom 120, the overall separation performance was similar across the column materials, with FWHM values ranging from 1.6 to 2.0 s, corresponding to Pc of 223 and 179, respectively (Figure 4B). The Kinetex column was an exception, exhibiting a slightly higher FWHM of 2.5 s (Pc = 146). Notably, peptides separated by ODS-IIIE and SOLAD columns exhibited a FWHM of 1.8 and 1.6 s, respectively, significantly narrower than those observed with the Reprosil and Kinetex columns. The longer gradient of Zoom 80, as expected, resulted in higher FWHM values relative to Zoom 120. Under these conditions, ODS-IIIE, SOLAD, and Luna exhibited significantly lower FWHM values (2.9, 2.6, and 2.6 s, respectively) with corresponding Pcs exceeding 200, indicating superior performance compared to Reprosil (3.4 s, Pc = 171) and Kinetex (3.8 s, Pc = 153) columns. Based on this comparative analysis, the short 150 μm i.d. ODS-IIIE and SOLAD nonporous particle columns demonstrate competitive performance with currently popular materials commonly employed for high-throughput peptide separation under equivalent gradient and pressure conditions.

**Figure 4:**
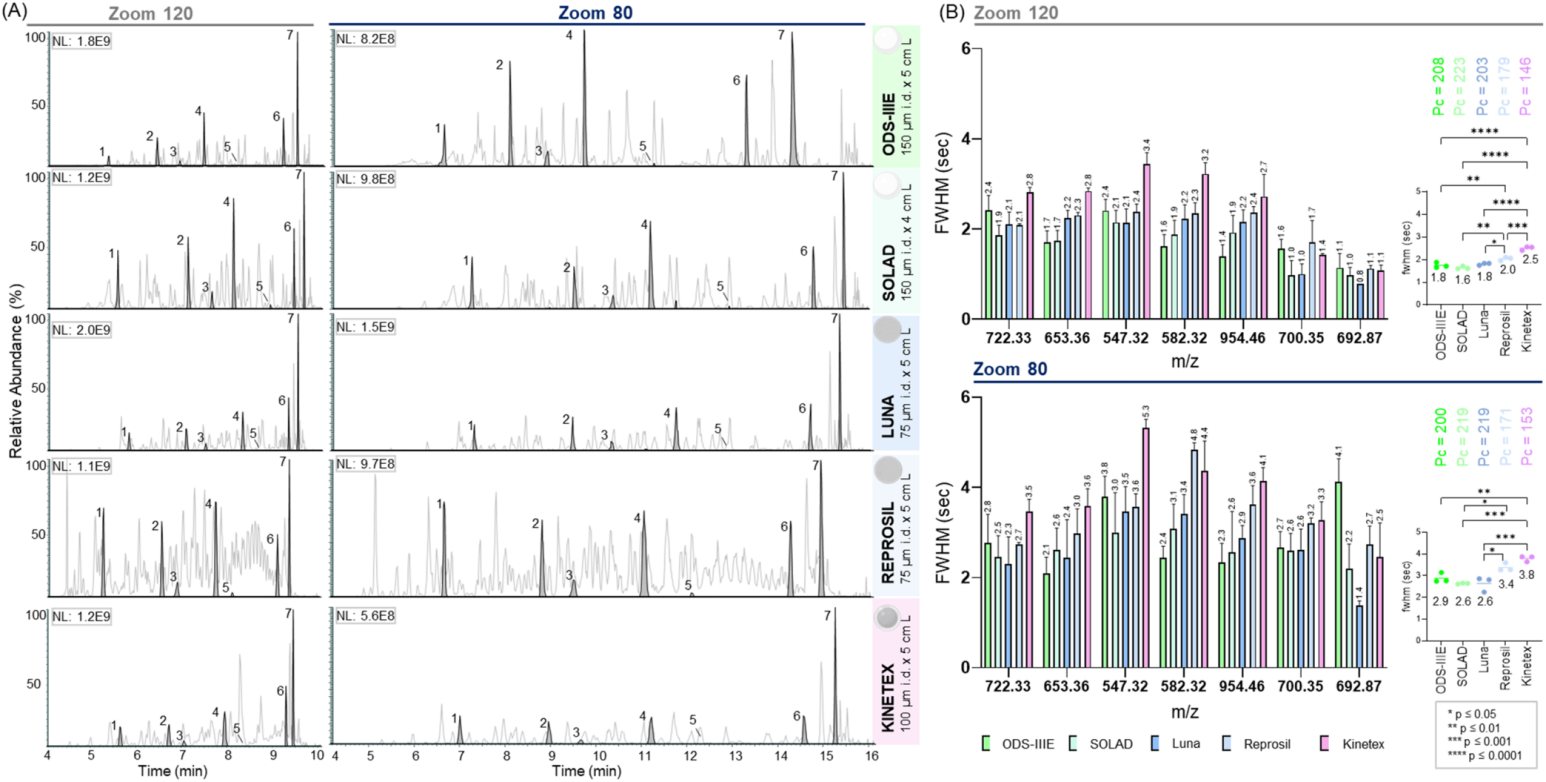
Performance of NPP, FPP, and SPP columns for high-throughput LC-MS/MS using an Evosep One LC system. (A) BPCs and XICs of selected tryptic peptides from a 10 fmol bovine serum albumin/α-casein digest separated using Whisper Zoom 120 (left) and Zoom 80 (right) methods. (B) Individual and average FWHM are presented to indicate the separation efficiency achieved with each column type, along with their respective peak capacity (Pc) values. Error bars show the standard deviation. Asterisks indicate statistically significant differences between columns. Mass spectrometry detection was performed using a Q Exactive.

### Evaluation of Peptide Loading Capacity for Short Nonporous C-18 Columns

While a common concern regarding nonporous materials is their potentially lower binding capacity compared to traditional porous particles,^23^ this limitation has become less critical with advancements in mass spectrometer sensitivity.^1,2^ Nevertheless, we also wanted to evaluate the loading capacity of the ODS-IIIE and SOLAD columns. To this end, we performed LC-MS/MS analyses of increasing amounts (0.25 to 50 ng) of Expi 293F digest with both Whisper Zoom methods and monitored the intensity and FWHM of three representative peptides, an early-eluting species, a mid-eluting species, and a late-eluting species, as proxies for determining the optimal loading capacity before column performance is compromised by saturation effects. It is well established that mass overloading can lead to peak shape distortion and signal saturation, ultimately compromising data quality and peptide identification.^38,39^ The loading capacity analysis (Figure S6 and Table S3) revealed that for both NPP columns and gradient methods, signal intensity for all three monitored peptides began to plateau above approximately 25 ng, a trend consistent with the overall peak intensities observed in the corresponding BPCs shown in Figures S7 and S8. While early- and mid-eluting peptides showed minimal changes in peak width upon saturation, the late-eluting peptide exhibited a marked increase in FWHM when the injected mass exceeded 25 ng, consistent with the observation that the overloading effects intensify with the increase in the retention factor *k*.^40^ Based on these trends, a loading amount below 25 ng of the human cell lysate digest appears to be optimal for both the ODS-IIIE and SOLAD columns under both gradient methods to preserve peak shape and prevent significant intensity saturation. Such amounts are compatible and optimal with most modern mass spectrometers.

### Performance of Nonporous and Porous Columns in High-Throughput DIA LC-MS/MS

Having established the peptide loading capacity of short ODS-IIIE and SOLAD columns, we proceeded to evaluate their performance against the other commonly utilized columns (Luna, Reprosil, and Kinetex) in a high-throughput DIA workflow using the Whisper Zoom 120 and 80 methods. To ensure a fair comparison, we selected a loading amount of 20 ng of Expi 293F digest, below the established saturation threshold observed for the short NPP columns. Using the Zoom 120 method, precursor identification was relatively consistent across the five columns, ranging from 19,740 (Kinetex) to 23,100 (SOLAD). The results were highly reproducible across technical replicates, as indicated by standard deviations below 3% for all columns (Figure 5 and Table S4). Similar numbers of precursors were identified using SOLAD, Reprosil, Luna, and ODS-IIIE. Kinetex showed the lowest precursor count, which was significantly lower compared to the other columns. Protein identification ranged from 2707 to 3130. Notably, nonporous particle columns (ODS-IIIE and SOLAD) yielded the highest numbers of proteins (>3000), followed by the fully porous particle columns, Luna (2942) and Reprosil (2977). The lowest protein count was observed for the superficially porous Kinetex column (2707), which was significantly lower than those obtained with the other columns. As expected, the longer gradient provided by the Zoom 80 method significantly increased both precursor and protein identification, with average improvements of 40 and 20%, respectively, relative to Zoom 120. Precursor counts with Zoom 80 ranged from 27,353 (Kinetex) to 32,813 (SOLAD), while protein IDs ranged from 3327 (Kinetex) to 3881 (ODS-IIIE). The same trend in precursor and protein identification performances observed with the Zoom 120 method was also seen with the Zoom 80 method. Based on these observations, both precursor and protein identification were consistent and reproducible for all columns tested across both gradient methods. The nonporous ODS-IIIE and SOLAD columns demonstrated comparable or superior performance to the fully and superficially porous columns using a high-throughput DIA workflow.

**Figure 5:**
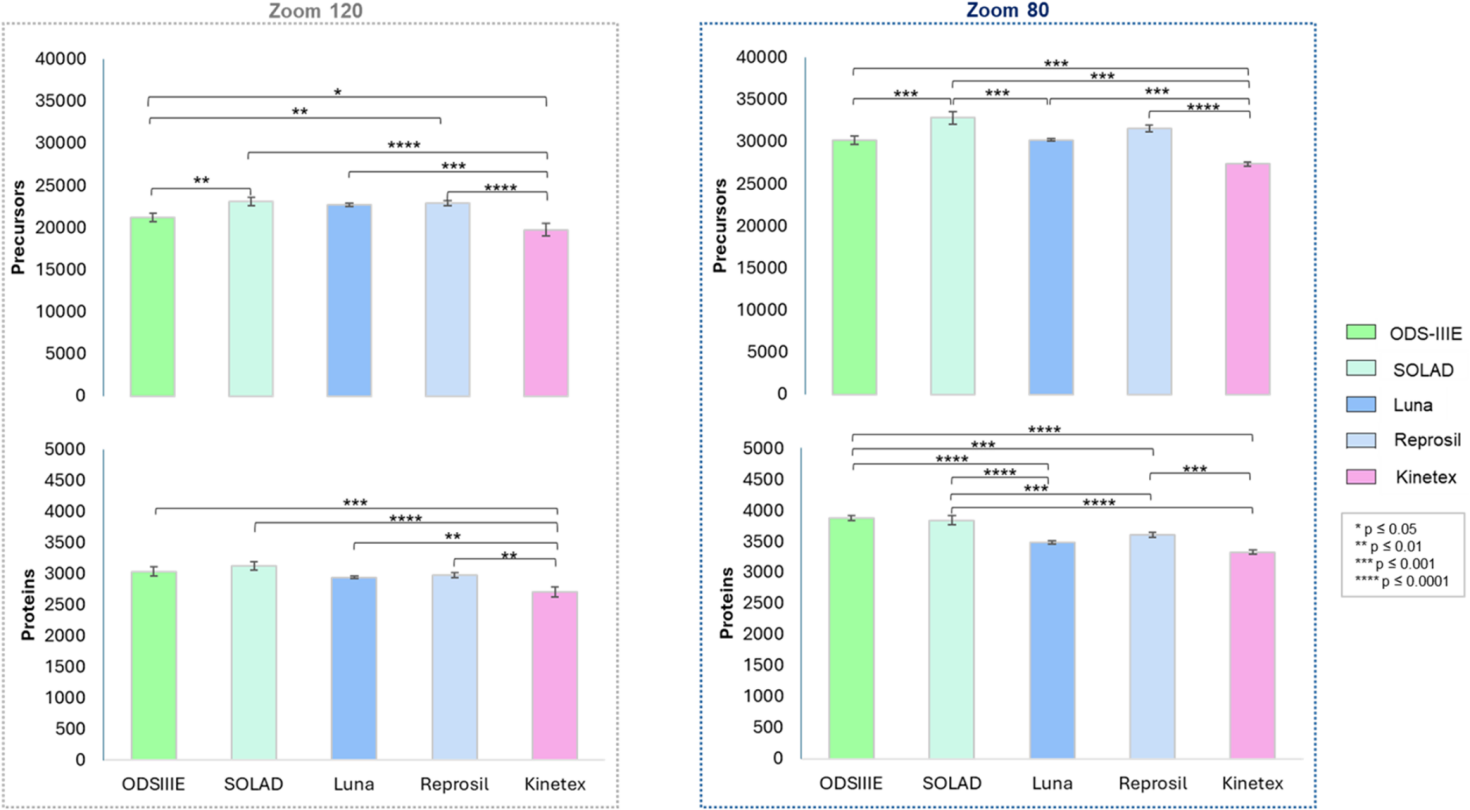
Evaluation of precursor and protein identification performance in the analysis of 20 ng of Expi 293F digest using the Evosep One LC system with Zoom 120 and Zoom 80 methods, coupled with DIA on an Orbitrap Exploris 480 mass spectrometer. The average number of precursors and proteins is represented by the bars, and error bars show the standard deviation calculated from three independent LC-MS/MS analyses. Asterisks indicate statistically significant differences in the number of identified precursors and proteins.

## Conclusions

We demonstrate that short nonporous C-18 columns packed with sub-2 μm particles offer competitive chromatographic performance for high-speed nanoLC-based proteomics workflows. We show that the long and short versions of these columns enable sharp peak profiles and efficient peptide separations within pressure limits compatible with widely used nanoLC systems. Comparative analyses reveal that nonporous columns deliver similar or superior separation performance relative to established fully and superficially porous C-18 materials while maintaining competitive protein/precursor identifications under high-throughput DIA conditions using 20 ng of cell lysate digest. Limited sample loading and poor retention/resolution of hydrophilic peptides does potentially limit the use of these columns for circumstances such as the analysis of a high dynamic range sample, which requires higher sample loads or applications requiring comprehensive coverage of specific proteins or PTMs, where some peptides will inevitably fall in the hydrophilic range poorly handled by such columns. However, nonporous columns are well-suited for complex proteome analysis, where the complexity of the sample far exceeds LC-MS performance and would make a useful component for high-throughput full proteome experiments. These findings highlight the potential of nonporous C-18 phases as effective alternatives for rapid, sensitive analyses of highly complex proteomes using short gradients and low sample input conditions.

## Supplementary Material











## Notes

Protein digestion and sample preparation; column packing, nano-liquid chromatography and mass spectrometry; raw data analysis (Method); resulting inner (i.d.) and outer (o.d.) diameters of laser-pulled tips fabricated from 75, 100, and 150 μm i.d. capillaries (Figure S1); base-peak chromatograms (BPCs) of a 10 fmol tryptic digest of bovine serum albumin/α-casein separated at increasing flow rates using an Ultimate 3000 RSLCnano system with a 150 μm i.d. × 25 cm ODS-IIIE column and a 15 min linear gradient (Figure S2); BPCs of a 10 fmol tryptic digest of bovine serum albumin/α-casein separated at increasing flow rates using an Ultimate 3000 RSLCnano system with a 150 μm i.d. × 15 cm SOLAD column and a 15 min linear gradient (Figure S3); BPCs and extracted ion chromatograms (XICs) of selected peptides from a 10 fmol bovine serum albumin/α-casein tryptic digest, analyzed by 15 min gradient LC-MS/MS at a flow rate of 200 nL/min using long and short ODS-IIIE and SOLAD columns (Figure S4); BPCs and high-pressure pump profiles from LC-MS/MS analysis of 10 fmol tryptic peptides derived from a bovine serum albumin/α-casein digest, separated using the Whisper Zoom 120 and Zoom 80 methods with nonporous (NPP), fully porous (FPP), and superficially porous particle (SPP) columns (Figure S5); peptide loading capacity evaluation of 150 μm i.d. × 5 cm L ODS-IIIE and 150 μm i.d. × 4 cm L SOLAD columns using Whisper Zoom 120 and Zoom 80 methods on Evosep One system (Figure S6); representative BPCs obtained from LC-MS/MS analysis of increasing amounts of Expi 293F digest (0.25–50 ng) using the Whisper Zoom 120 and Zoom 80 methods on an Evosep LC system equipped with a 150 μm i.d. × 5 cm L ODS-IIIE column (Figure S7); representative base-peak chromatograms obtained from LC-MS/MS analysis of increasing amounts of Expi 293F digest (0.25–50 ng) using the Whisper Zoom 120 and Zoom 80 gradient methods on an Evosep LC system equipped with a 150 μm i.d. × 4 cm L SOLAD column (Figure S8) (PDF)
Temperature Flow rate NPP rev (Table S1) (XLSX)
FWHM all columns rev (Table S2) (XLSX)
Loading capacity ODSIII Solad rev (Table S3) (XLSX)
DIA all columns rev (Table S4) (XLSX)
