# Supplementary material for: Are Solid Particles Ready for Prime-Time Proteomics?
Source: Anal Chem. 2025 Aug 20;97(34):18681–8. doi: 10.1021/acs.analchem.5c03073 (PMC12409696; doi:10.1021/acs.analchem.5c03073)
Supplement: Supplementary file 1 [file ac5c03073_si_001.pdf]

Supporting Information

Are Solid Particles Ready for Prime-Time Proteomics?

Eduardo S. Kitano[1,2], Yana Demyanenko[1,2], Shabaz Mohammed[1,3,4*]

[1] Rosalind Franklin Institute, Harwell Campus, OX11 0QX Didcot, United Kingdom

[2] Department of Pharmacology, University of Oxford, OX1 3QT Oxford, United Kingdom

[3] Department of Biochemistry, University of Oxford, OX1 3QU, Oxford, United Kingdom

[4] Department of Chemistry, University of Oxford, OX1 3TA, Oxford, 16 United Kingdom

*Corresponding author: shabaz.mohammed@rfi.ac.uk

Table of Contents

METHOD: Protein Digestion and Sample Preparation; Column Packing, Nano-Liquid Chromatography and Mass Spectrometry; Raw Data Analysis.

FIGURE S1. Resulting inner (i.d.) and outer (o.d.) diameters of laser-pulled tips fabricated from 75, 100, and 150 µm i.d. capillaries. Capillaries (n = 3 per type) were pulled using a Sutter P-2000 laser puller.

FIGURE S2: Base-peak chromatograms (BPCs) of a 10 fmol tryptic digest of bovine serum albumin/α-casein separated at increasing flow rates using an Ultimate 3000 RSLCnano system with a 150 µm i.d. × 25 cm ODS-IIIE column and a 15-minute linear gradient (7-55% solvent B).

FIGURE S3: Base-peak chromatograms (BPCs) of a 10 fmol tryptic digest of bovine serum albumin/α-casein separated at increasing flow rates using an Ultimate 3000 RSLCnano system with a 150 µm i.d. × 15 cm SOLAD column and a 15-minute linear gradient (7-55% solvent B).

FIGURE S4: Base-peak (BPCs) and extracted ion chromatograms (XICs) of selected peptides from a 10 fmol bovine serum albumin/α-casein tryptic digest, analysed by 15-min gradient LC-MS/MS at a flow rate of 200 nL/min using long and short ODS-IIIE and SOLAD columns.

FIGURE S5: Base-peak chromatograms (BPCs) and high-pressure pump profiles from LC-MS/MS analysis of 10 fmol tryptic peptides derived from a bovine serum albumin/α-casein digest, separated using the Whisper Zoom 120 and Zoom 80 methods with nonporous (NPP), fully porous (FPP), and superficially porous particle (SPP) columns.

FIGURE S6: Peptide loading capacity evaluation of 150 µm i.d. × 5 cm L ODS-IIIE and 150 µm i.d. × 4 cm L SOLAD columns using Whisper Zoom 120 and Zoom 80 methods on Evosep One system.

FIGURE S7: Representative base peak chromatograms obtained from LC-MS/MS analysis of increasing amounts of Expi 293F digest (0.25 – 50 ng) using the Whisper Zoom 120 and Zoom 80 methods on an Evosep LC system equipped with a 150 µm i.d. × 5 cm L ODS-IIIE column.

FIGURE S8: Representative base peak chromatograms obtained from LC-MS/MS analysis of increasing amounts of Expi 293F digest (0.25 – 50 ng) using the Whisper Zoom 120 and Zoom 80 gradient methods on an Evosep LC system equipped with a 150 µm i.d. × 4 cm L SOLAD column.

METHOD

Protein Digestion and Sample Preparation. Bovine serum albumin (BSA, Sigma) was solubilized in 1 M urea, 100 mM ammonium bicarbonate (AmBic), while α-casein (Sigma) was dissolved in 8 M urea, 100 mM AmBic. BSA and casein samples were reduced with 10 mM tris(2-carboxyethyl)phosphine (TCEP) for 5 minutes at room temperature and alkylated with 50 mM 2-chloroacetamide (CAA, Sigma) for 30 minutes in the dark. The casein sample was then diluted with 100 mM AmBic to a final urea concentration of 4 M. Both BSA and casein samples were subsequently digested with Lys-C (Wako, biochemistry grade) with a protein-to-enzyme ratio of 100:1 (w/w) for 2 hours at 37 °C. Following Lys-C digestion, the samples were further diluted with 100 mM AmBic to achieve final urea concentration of approximately 1 M for casein and 0.25 M for BSA. Trypsin digestion (Promega) was performed at 37 °C using enzyme-to-substrate ratios of 1:30 (casein) and 1:40 (BSA), with an incubation time of 4 hours. After digestion, samples were acidified with formic acid (FA) and cleaned up using Oasis® HLB cartridges (3 cc, 60 mg sorbent, Waters). Eluted peptides were dried using a Genevac concentrator. Casein and BSA digests were mixed and diluted in either 5% FA / 5% dimethyl sulfoxide (DMSO) to a final concentration of 10 fmol/µL for nanoUPLC analyses, or with 5% FA / 0.015% n-Dodecyl-β-D-maltoside (DDM) to a final concentration of 2 fmol/µL prior to loading onto Evotip pure (Evosep Biosystems) for subsequent nanoHPLC analysis.

Expi293F cells (Gibco) were resuspended in a lysis buffer composed of 8 M urea, 100 mM AmBic, and a protease inhibitor cocktail (Roche). Samples were sonicated at 4 °C using a Bioruptor Ultrasonicator (Bioruptor Pico, Diagenode) set to ultra-low frequency for 30 cycles of 30 seconds each. After centrifugation at 14,000 x g, 4 °C for 15 minutes, the supernatants were collected, and the protein concentrations were

estimated by BCA assay (Pierce). Proteins were reduced with 10 mM TCEP for 30 minutes at room temperature followed by alkylation with 50 mM CAA for 30 minutes in the dark. The urea concentration was diluted to 4 M with 100 mM AmBic, and the first step of digestion was carried out with Lys-C protease for 2 hours at 37 °C with a protein-to-enzyme ratio of 50:1 (w/w). Samples were further diluted to 1.3 M urea and digested with trypsin for 4 hours at 37 °C with a protein-to-enzyme ratio of 50:1 (w/w). Peptide samples were cooled to 4 °C, acidified with FA to a final concentration of 5% (v/v) and centrifuged at 16,200 × g for 10 minutes to remove any precipitate. The supernatants were diluted in 5% FA / 0.015% DDM at concentrations ranging from 0.0125 to 2.5 ng/µL prior to loading onto Evotip pure for subsequent nanoHPLC analysis.

Peptide samples analysed using the Evosep One HPLC system (Evosep Biosystems) were prepared with Evotips following the manufacturer's instructions.

Column Packing. Nonporous (NPP), fully porous (FPP), and superficially porous C-18 particles (SPP) were prepared as slurries at a concentration of 100 mg/mL. FPP Reprosil Gold™, and the NPPs ODS-IIIE™ and SOLAD™ were slurried in 100% acetone, while Luna Omega Polar™ (FPP) and Kinetex® (SPP) were slurried in 100% methanol. Prior to packing, the materials were washed three times with either acetone or methanol, followed by 20 minutes of sonication and particle sedimentation. Fused silica capillaries (Polymicro Technologies) with internal diameters (i.d.) of 75, 100 and 150 µm, and outer diameter (o.d.) of 360 µm were pulled using a P-2000 laser puller (Sutter Instrument). Laser parameters were set as follows: HEAT = 245, FIL = -, VEL = 15, DEL = 132, PUL = - for the 75 µm i.d. capillaries, and HEAT = 300, FIL = -, VEL = 15, DEL = 255, PUL = 10 for the 100 and 150 µm i.d. capillaries. Capillaries were then packed with the respective C-18 materials using a PC8500 Pressure Injection Cell (Next Advance), following the protocol described by Kovalchuck[1], with the production of a self-assembled particles as a frit at the outlet of the columns.[2] Seventy-five micrometre i.d. capillaries were packed with fully porous Reprosil Gold™ (1.9 µm, Dr. Maisch) and Luna Omega Polar™ (1.6 µm, Phenomenex); 100 µm i.d. capillary was packed with superficially porous Kinetex® [1.7 µm – 1.25 µm (core) and 0.23 µm (shell thickness) – Phenomenex)]; and 150 µm i.d. capillaries, with nonporous ODS-IIIE™ (1.5 µm, Eprogen/Promigen Life Sciences) and SOLAD™ (1.0 µm, Glantreo). Columns were

packed at 120 bar for a minimum of 2 hours, after which the pressure was gently released. To consolidate the column beds, a constant flow of 70% acetonitrile (ACN) was applied at 800 bar for 2 hours using an NCS-3500RS nano pump (Thermo Scientific). Following consolidation, columns were cut to the desired lengths: 25 cm and 5 cm for ODS-IIIE; 15 cm and 4 cm for SOLAD; and 5 cm for Luna, Reprosil, and Kinetex. A sol-gel frit was formed at the column inlet according to the method described by Maiolica.[3] Briefly, the capillary inlets were gently pushed on a glass microfibre filter (GF/C, Whatman) previously wet with a 1:1 (v/v) mixture of Kasil 1624 (Next Advance) and 25% formamide. The frits were then polymerized at 85 °C for 16 hours. LC-MS/MS analyses were performed using three independent 5-cm ODS-IIIE and 4-cm SOLAD columns, while experiments involving the longer NPP columns and the 5-cm Luna, Reprosil, and Kinetex columns, were carried out using a single column of each type.

Nano-Liquid Chromatography and Mass Spectrometry. To optimize flow rates and column temperatures for the NPP C-18 columns, 10 fmol BSA/casein digest was analysed using an Ultimate 3000 RSLCnano system (Thermo Fisher Scientific) equipped with a C-18 PepMap100 trap column (300 µm i.d. x 5 mm L, 100Å, Thermo Fisher Scientific), and separated on 25-cm ODS-IIIE and 15-cm SOLAD analytical columns. Solvent A was composed of 5% DMSO and 0.1% formic acid in water, while solvent B contained 5% DMSO and 0.1% FA in ACN. Chromatographic separations were conducted over a temperature range of 30 – 70 °C at a constant flow rate of 200 nL/min, and at flow rates ranging from 50 to 500 nL/min at 40 °C (ODS-IIIE) and 50 °C (SOLAD), utilizing a 15-minute linear gradient as follows: a 5-minute hold at 2% B, and 7% to 55% solvent B over 15 minutes, followed by an increase to 99% B over 2 minutes, a 1-minute hold at 99% B, and re-equilibration to 2% B for variable duration depending on the employed flow rate. The BSA/casein digest was also subjected to a 15-minute separation using shorter ODSIII (5 cm) and SOLAD (4 cm) columns at 200 nL/min at 20 °C. To assess and compare the separation performance of ODS-IIIE and SOLAD columns with that of FPP (Luna and Reprosil) and SPP (Kinetex) columns, a 10 fmol BSA/casein digest was analysed using the predefined Whisper Zoom 120 (10-minute gradient) and Zoom 80 (16-minute gradient) methods on an Evosep One LC. Increasing amounts of Expi 293F digest (0.25 to 50 ng) separated by the Whisper Zoom 120 and Zoom 80

methods was used for the assessment of peptide loading capacity of 5-cm ODSIII and 4-cm SOLAD columns. Data-Dependent Acquisition (DDA) mass spectrometry was performed on a Q Exactive mass spectrometer (Thermo Fisher Scientific) using the following parameters: Full MS scans (350-1,400 m/z) were acquired in the Orbitrap at 70,000 resolution (at m/z 200) using an AGC target of 3×10^6 and a maximum injection time of 50 ms. The top five most intense precursors (charge states 2–7) were isolated with a 1.5 Th window and fragmented using higher-energy collisional dissociation (HCD) at a normalized collision energy (NCE) of 30%. MS/MS spectra were acquired in the Orbitrap at 17,500 resolution with an AGC target of 5×10^4 and a maximum injection time of 128 ms. Dynamic exclusion was enabled with a 5 s exclusion duration, ±10 ppm mass tolerance, and a repeat count of 1.

Twenty nanograms of Expi 293F digest were separated on 5-cm NPP (150 µm i.d.), FPP (75 µm i.d.), and SPP (100 µm i.d.) columns, except for the SOLAD material, where a 4 cm column was used. Separations were carried out using the Whisper Zoom 120 and Zoom 80 methods on an Evosep One system coupled to an Orbitrap Exploris 480 mass spectrometer (Thermo Fisher Scientific) operating in DIA mode. Full MS scans (350−1400 m/z) were acquired at 60,000 resolution with a 300% normalised AGC target, and 25 ms injection time. DIA was performed using 33 isolation windows (12 m/z width) spanning the m/z range of 400–800. Precursor ions were fragmented using HCD with a NCE of 30%. MS/MS spectra were acquired in the Orbitrap at 15,000 resolution with a normalized AGC target of 2000% and injection time of 22 ms. The total cycle time was 3 seconds.

LC-MS/MS analyses were conducted in triplicate for each column, except for the column temperature evaluation, where a single analysis was performed per column at each tested temperature.

Raw Data Analysis. Full-width at half maximum (FWHM) and peptide intensities were manually calculated/obtained from the extracted ion chromatograms (XICs) of selected peptides from BSA/casein or Expi 293F digests using Freestyle™ software version 1.8.51.0 (Thermo Fisher Scientific). Peak capacity (Pc) was calculated according to the formula described by Kovalchuck et al.[1]

$$Pc = 1 + 0.589 * \frac{t}{FWHM}$$

Where t = full gradient time and FWHM = full width at half maximum. FWHM values were further processed using GraphpadPrism software (v. 10.4.2) and analysed using one-way analysis of variance (ANOVA) followed by Tukey's post hoc test to evaluate pairwise differences between groups.

DIA LC-MS/MS data were analysed by DIA-NN software[4] (version 1.8.1) using library-free search. Spectral library was predicted in silico from the Uniprot human proteome database (Proteome ID: UP000005640, downloaded in August 2022, 79,759 sequences). Methionine oxidation and cysteine carbamidomethylation were set as variable and fixed modification, respectively. Enzyme specificity was set to trypsin/P, allowing up to one missed cleavage, and peptide lengths were restricted to 7–30 amino acids. The following parameters were used: protein inference set to 'Genes', neural network classifier set to 'Single-pass mode', and quantification strategy selected as 'Robust LC (high precision)'. Cross-run normalization was performed using the 'RT-dependent' setting, and spectral library generation was enabled using the 'Smart profiling' mode. Speed and RAM usage were configured for 'Optimal results'. Mass accuracy settings for both precursor and fragment ions were set to 0 to enable automatic inference. The options 'No shared spectra', 'Heuristic protein inference', and 'Unrelated runs' were enabled. The false discovery rate (FDR) at the precursor and protein levels was estimated and filtered to 1%. ANOVA was used to determine whether there were statistically significant differences among the means of peptide/protein IDs. Raw data and search results have been deposited in the ProteomeXchange Consortium via the PRIDE[5] partner repository under the dataset identifier PXD064019.

Supplemental Figures

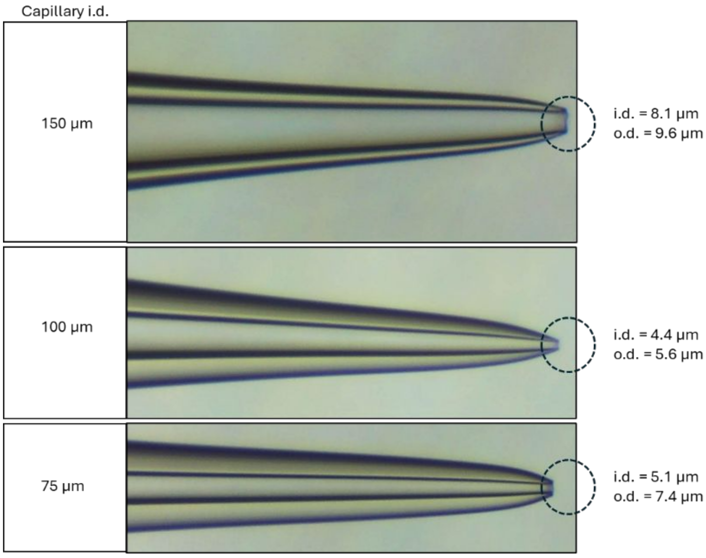

	Tip i.d.		
Replicate	75 µm	100 µm	150 µm
R1	5	4.4	8.6
R2	5.2	4.6	8.4
R3	5.2	4.3	7.3
Average i.d.	5.1	4.4	8.1
SD	0.1	0.2	0.7

	Tip o.d.		
Replicate	75 µm	100 µm	150 µm
R1	7.7	5.5	9.8
R2	7.1	5.4	10.4
R3	7.3	5.8	8.6
Average o.d.	7.4	5.6	9.6
SD	0.3	0.2	0.9

Figure S1. Resulting inner (i.d.) and outer (o.d.) diameters of laser-pulled tips fabricated from 75, 100, and 150 µm i.d. capillaries. Capillaries (n = 3 per type) were pulled using a Sutter P-2000 laser puller.

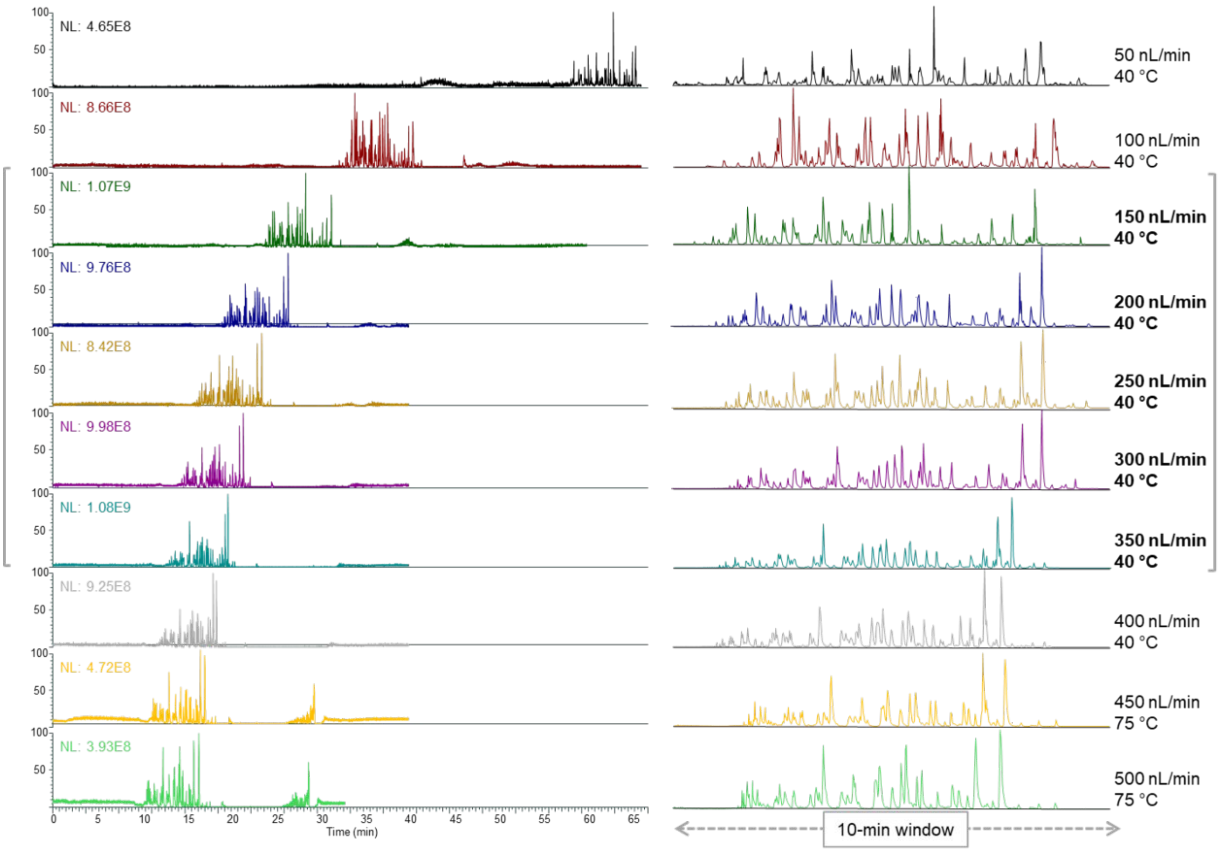

Figure S2. BPCs of a 10 fmol tryptic digest of bovine serum albumin/α-casein separated at increasing flow rates using an Ultimate 3000 RSLCnano system with a 150 µm i.d. × 25 cm ODS-IIIE column and a 15-minute linear gradient (7-55% solvent B). The column temperature was maintained at 40 °C during LC-MS/MS analysis and increased to 75 °C when the flow rate exceeded 400 nL/min to control backpressure. Optimal flow rates (150-350 nL/min) are highlighted in brackets. Detection was performed with a Q Exactive mass spectrometer.

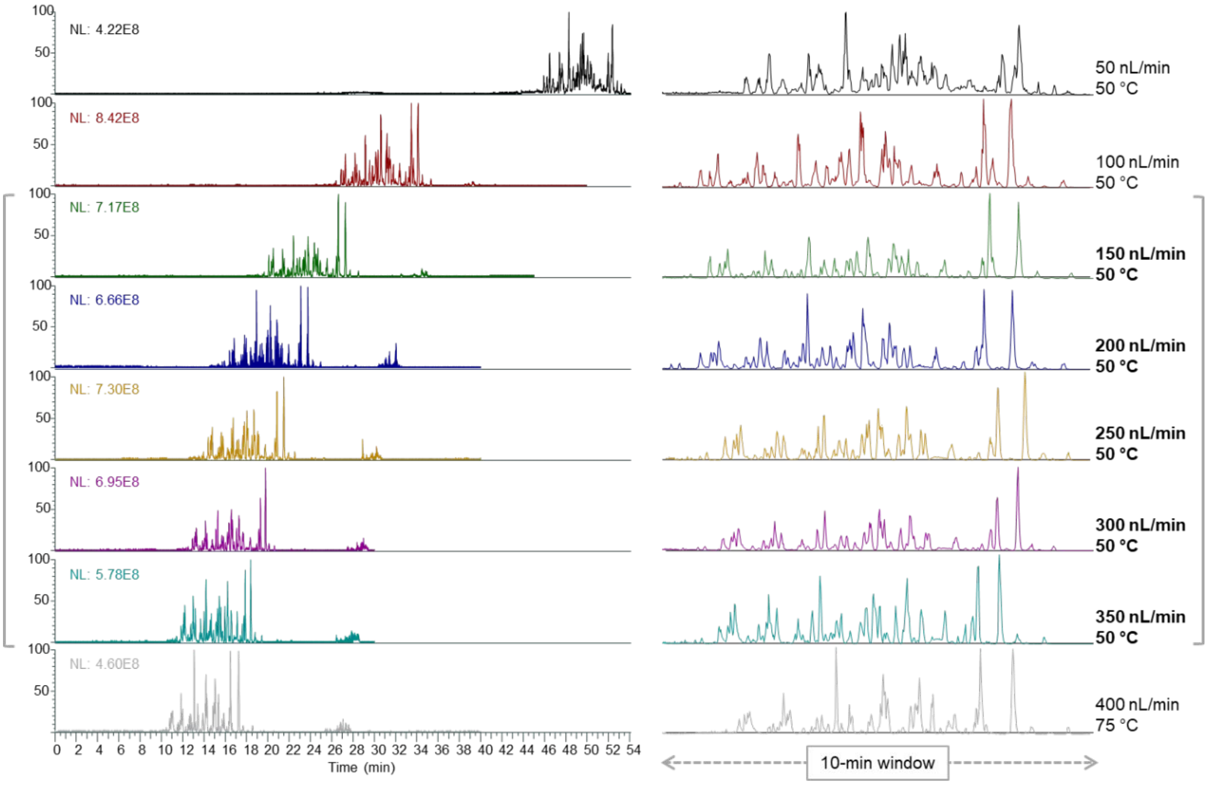

Figure S3. BPCs of a 10 fmol tryptic digest of bovine serum albumin/α-casein separated at increasing flow rates using an Ultimate 3000 RSLCnano system with a 150 µm i.d. × 15 cm SOLAD column and a 15-minute linear gradient (7-55% solvent B). The column temperature was maintained at 50 °C during LC-MS/MS analysis and increased to 75 °C when the flow rate exceeded 350 nL/min to control backpressure. Optimal flow rates (150-350 nL/min) are highlighted in brackets. Detection was performed with a Q Exactive mass spectrometer.

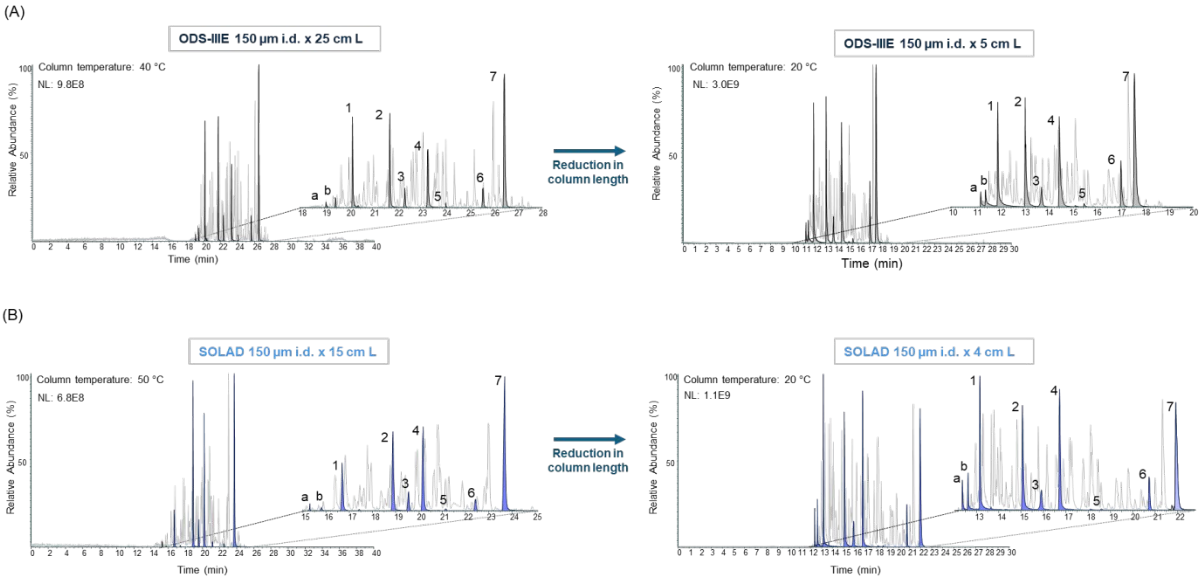

Figure S4. BPCs and XICs of selected peptides from a 10 fmol bovine serum albumin/α-casein tryptic digest, analysed by 15-min gradient LC-MS/MS at a flow rate of 200 nL/min using long and short ODS-IIIE (A) and SOLAD (B) columns. LC-MS/MS analysis using ‘long’ columns was performed on an Ultimate 3000 RSLCnano system coupled to a Q Exactive, while analysis with the short columns was carried out on an Orbitrap Exploris 480. Early-eluting peptides (m/z 488.54 and 625.78) are represented by peaks "a" and "b", respectively.

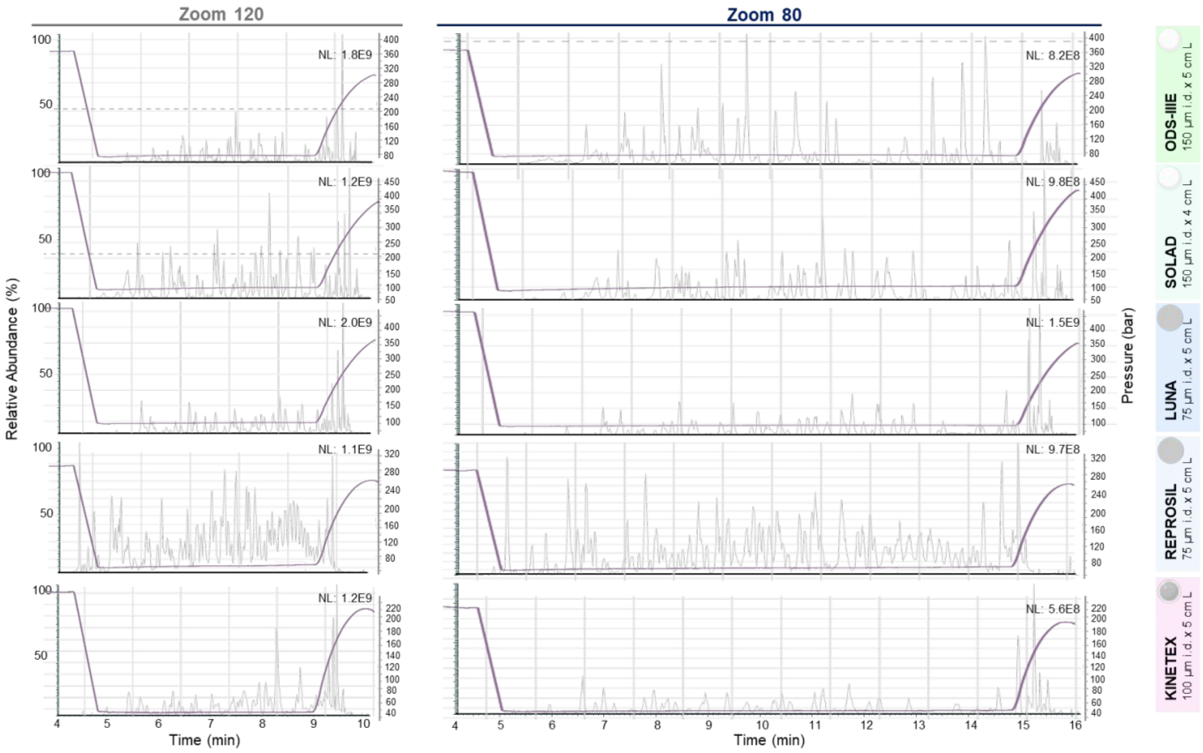

Figure S5. BPCs and high-pressure pump profiles from LC-MS/MS analysis of 10 fmol tryptic peptides derived from a bovine serum albumin/α-casein digest, separated using the Whisper Zoom 120 (left) and Zoom 80 (right) methods with NPP, FPP, and SPP columns.

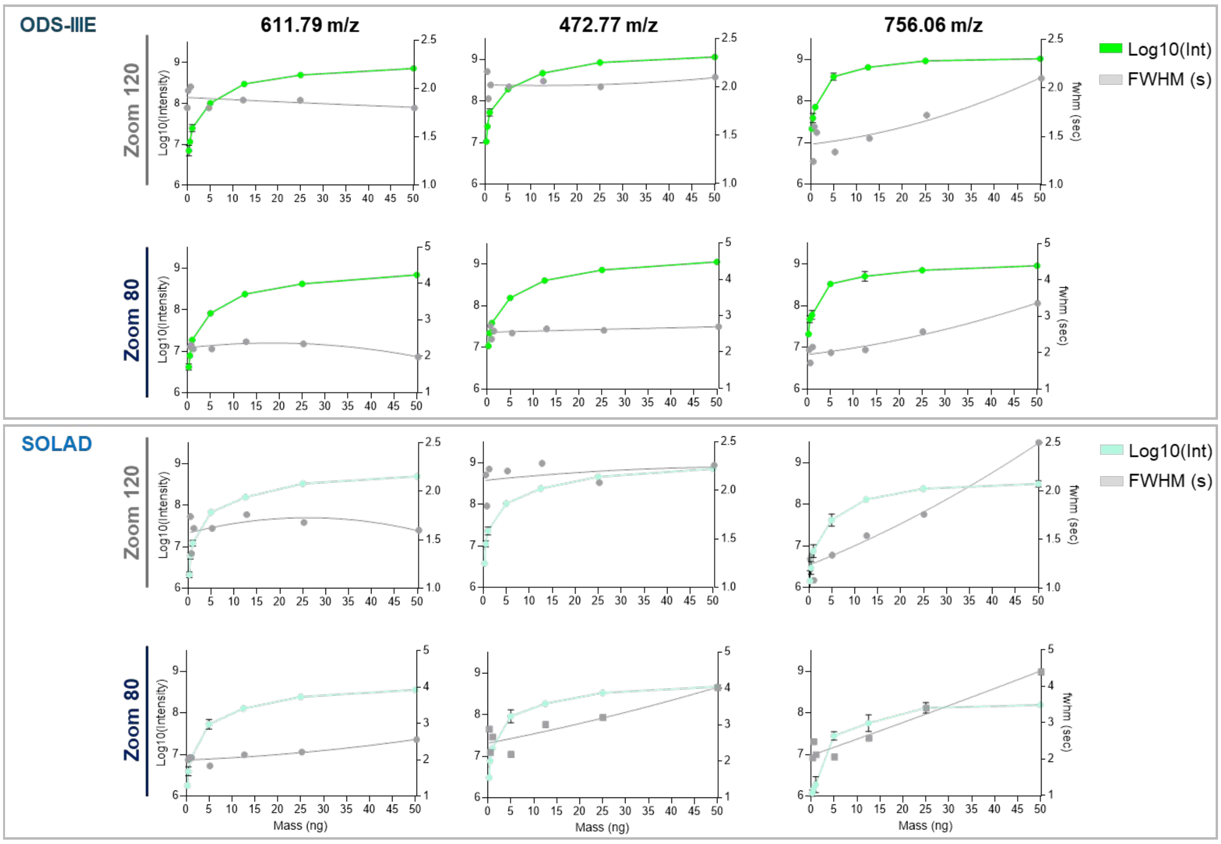

Figure S6. Peptide loading capacity evaluation of 150 µm i.d. × 5 cm L ODS-IIIE (top panel) and 150 µm i.d. × 4 cm L SOLAD (bottom panel) columns. Average intensities and FWHM (n=3) of three peptides are plotted against increasing amounts of Expi 293F digest (0.25 to 50 ng) separated by Whisper Zoom 120 and Zoom 80 methods on an Evosep One LC system. Grey lines indicate second-degree polynomial fits applied to the data points to visualize trends in FWHM. Error bars correspond to the standard deviation.

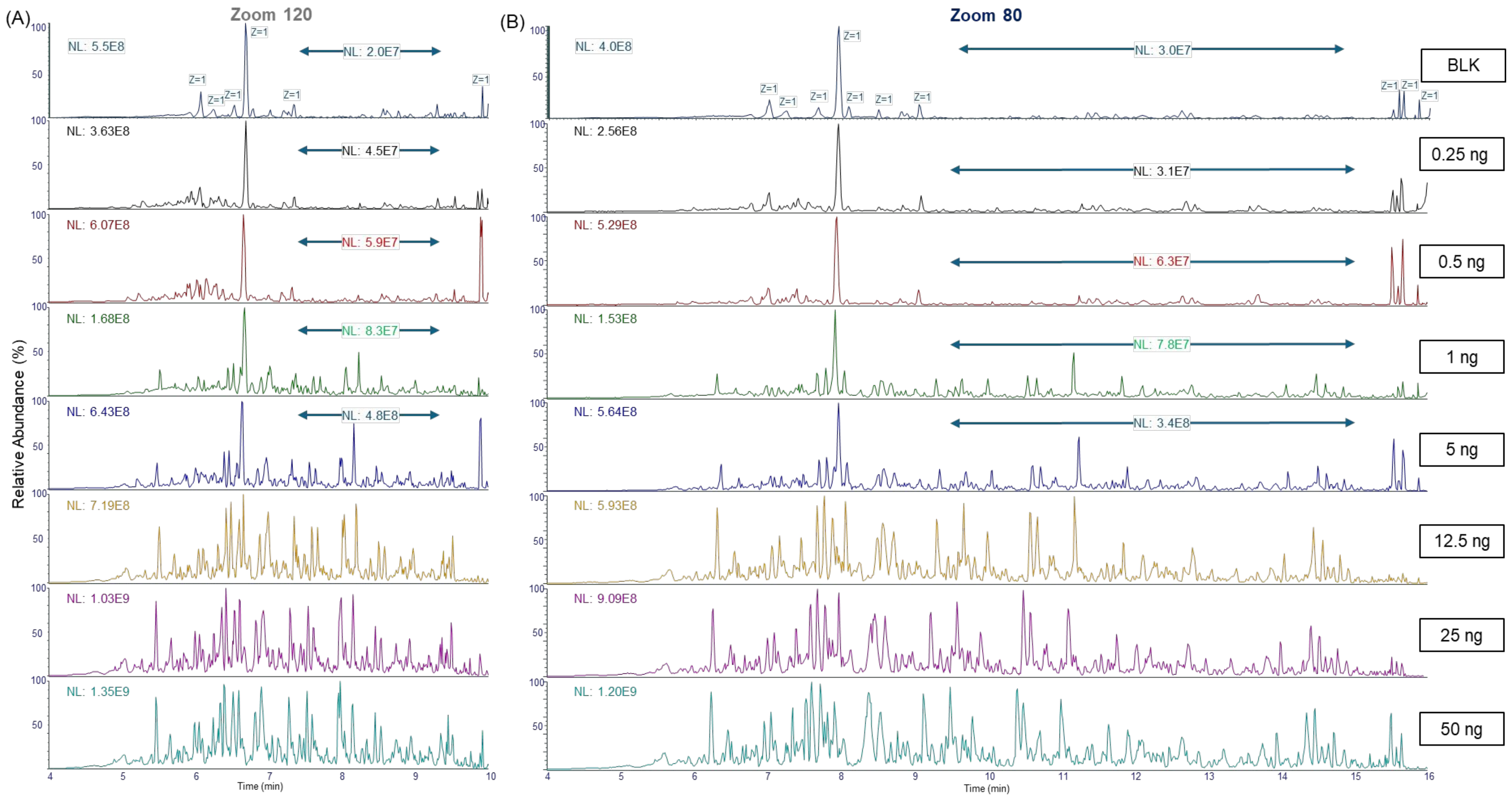

Figure S7. Representative base peak chromatograms obtained from LC-MS/MS analysis of increasing amounts of Expi 293F digest (0.25 – 50 ng), as well as a blank (BLK) sample (resuspension buffer only), using the Whisper Zoom 120 (A) and Zoom 80 (B) gradient methods on an Evosep LC system equipped with a 150 µm i.d. × 5 cm L ODS-IIIE column. Double-arrow lines indicate regions that are magnified to provide a clearer visualization of the average peptide intensities. Mass spectrometry detection was performed using a Q Exactive system.

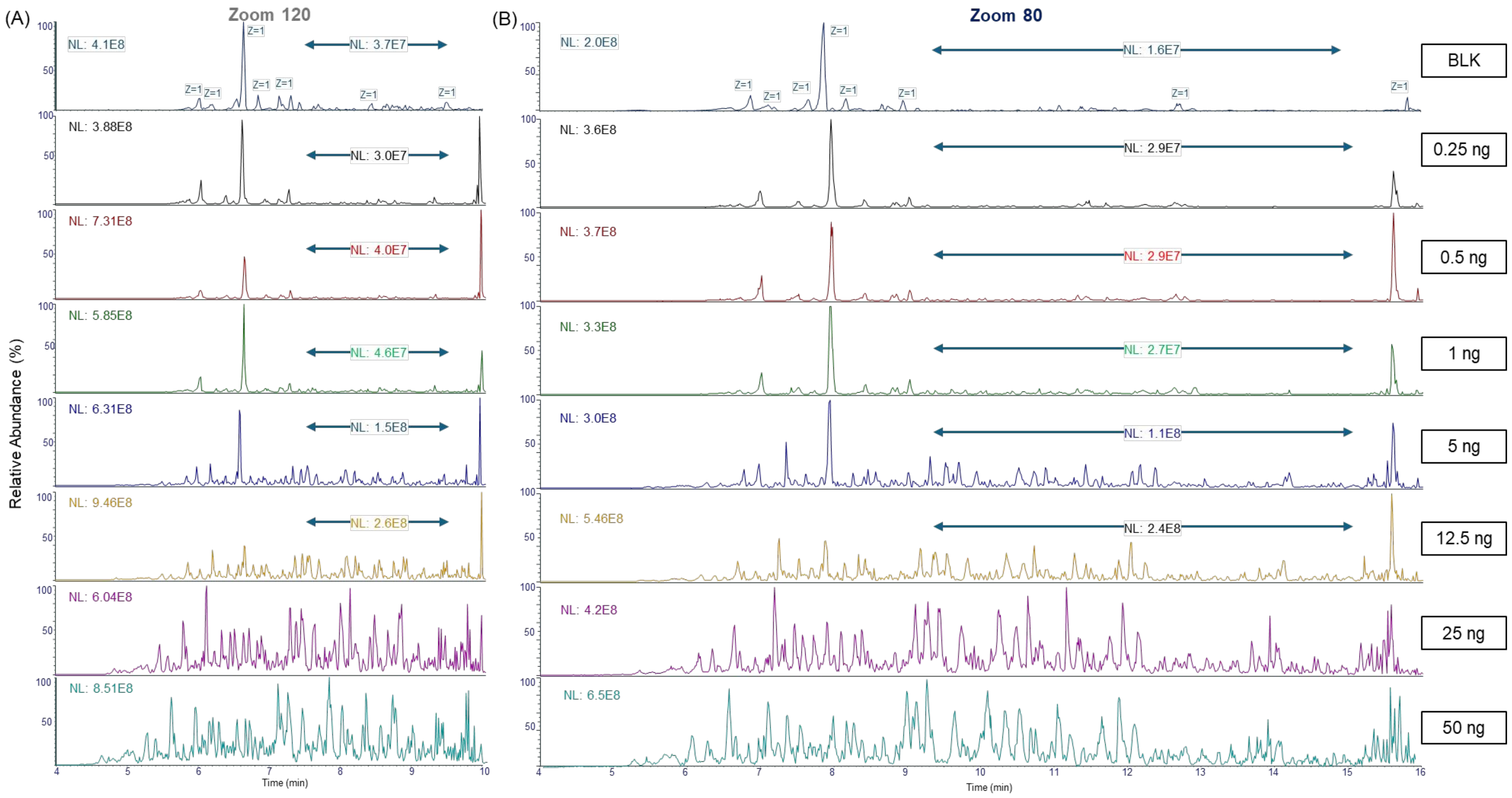

Figure S8. Representative base peak chromatograms obtained from LC-MS/MS analysis of increasing amounts of Expi 293F digest (0.25 – 50 ng), as well as a blank (BLK) sample (resuspension buffer only), using the Whisper Zoom 120 (A) and Zoom 80 (B) gradient methods on an Evosep LC system equipped with a 150 μm i.d. × 4 cm L SOLAD column. Double-arrow lines indicate regions that are magnified to provide a clearer visualization of the average peptide intensities. Mass spectrometry detection was performed using a Q Exactive system.

References

(1) Kovalchuk, S. I.; Jensen, O. N.; Rogowska-Wrzesinska, A. FlashPack: Fast and Simple Preparation of Ultrahigh-performance Capillary Columns for LC-MS. *Mol Cell Proteomics* **2019**, *18* (2), 383-390. DOI: 10.1074/mcp.TIR118.000953 From NLM.
(2) Ishihama, Y.; Rappsilber, J.; Andersen, J. S.; Mann, M. Microcolumns with self-assembled particle frits for proteomics. *Journal of Chromatography A* **2002**, *979* (1), 233-239. DOI: https://doi.org/10.1016/S0021-9673(02)01402-4.
(3) Maiolica, A.; Borsotti, D.; Rappsilber, J. Self-made frits for nanoscale columns in proteomics. *Proteomics* **2005**, *5* (15), 3847-3850. DOI: 10.1002/pmic.200402010 From NLM.
(4) Demichev, V.; Messner, C. B.; Vernardis, S. I.; Lilley, K. S.; Ralser, M. DIA-NN: neural networks and interference correction enable deep proteome coverage in high throughput. *Nature Methods* **2020**, *17* (1), 41-44. DOI: 10.1038/s41592-019-0638-x.
(5) Perez-Riverol, Y.; Bai, J.; Bandla, C.; García-Seisdedos, D.; Hewapathirana, S.; Kamatchinathan, S.; Kundu, D. J.; Prakash, A.; Frericks-Zipper, A.; Eisenacher, M.; et al. The PRIDE database resources in 2022: a hub for mass spectrometry-based proteomics evidences. *Nucleic Acids Res* **2022**, *50* (D1), D543-d552. DOI: 10.1093/nar/gkab1038 From NLM.
